# Is There an Association between Work Stress and Diurnal Cortisol Patterns? Findings from the Whitehall II Study

**DOI:** 10.1371/journal.pone.0081020

**Published:** 2013-12-03

**Authors:** Jing Liao, Eric J. Brunner, Meena Kumari

**Affiliations:** Department of Epidemiology and Public Health, University College London, London, United Kingdom; University of Lübeck, Germany

## Abstract

**Objective:**

The evidence on whether there is work stress related dysregulation of the hypothalamic-pituitary-adrenal axis is equivocal. This study assessed the relation between work stress and diurnal cortisol rhythm in a large-scale occupational cohort, the Whitehall II study.

**Methods:**

Work stress was assessed in two ways, using the job-demand-control (JDC) and the effort-reward-imbalance (ERI) models. Salivary cortisol samples were collected six times over a normal day in 2002–2004. The cortisol awakening response (CAR) and diurnal cortisol decline (slope) were calculated.

**Results:**

In this large occupational cohort (N = 2,126, mean age 57.1), modest differences in cortisol patterns were found for ERI models only, showing lower reward (β = −0.001, P-value = 0.04) and higher ERI (β = 0.002, P-value = 0.05) were related to a flatter slope in cortisol across the day. Meanwhile, moderate gender interactions were observed regarding CAR and JDC model.

**Conclusions:**

We conclude that the associations of work stress with cortisol are modest, with associations apparent for ERI model rather than JDC model.

## Introduction

Work stress has been established as a risk factor for a range of health impairments, particularly cardiovascular disease [1], metabolic syndrome [2], [3] and Type 2 diabetes mellitus [4], [5]. Two dominant work stress models have been widely employed in these analyses: the job demand control (JDC) model [6] and the effort-reward imbalance (ERI) model [7]. The JDC model postulates a combination of lower control (less skill utilization and lower decision authority) and higher work demand (more quantitative work load and conflicting demands) will trigger job strain; whereas the ERI model emphasizes social reciprocity, such that a sustained unfair trade-off between effort (cost) and reward (gain) will elicit negative emotions and further lead to adverse long-term health consequences. Similarities exist between these two models as both of them tap psychosocial disequilibrium [8] and highly correlated items are adopted in respective scales [9]. Nevertheless, there are distinctive conceptual and methodological differences as the JDC model refers to the structural characteristics of the psychosocial environment at work, emphasizing the power structure, labour division and workplace democracy [10]; in contrast, the ERI model takes personal coping strategy into account and highlights perceptions of reciprocity, embodied by wage, esteem and job security [11], [12].

The hypothalamic-pituitary-adrenal (HPA) axis, one of the main axes of neuroendocrine stress response, is hypothesized as a pathway by which work stress might be related to adverse health outcomes. However, there is inconsistent evidence on work stress related dysregulation of the HPA axis. In terms of the JDC model, job strain has been associated with raised morning cortisol [13]–[16] and increased cortisol secretion across the day [17]; whereas inverse or no significant relations have also been reported [18]–[20]. Similarly, a mixed picture emerges regarding the ERI model: some studies reported a blunted cortisol response in relation to ERI [14], [21], [22], yet other two studies [23], [24] did not observe any significant association. Evidence to date has been synthesized in two recent reviews [25], [26]. Chida and Steptoe (2009) reported that the cortisol awakening response (CAR), a rapid rise in cortisol levels following wakening, was weakly but positively associated with work stress. In this review, 4 studies included [13], [23], [27], [28] examined the JDC model (average size 159), and 2 studies [23], [27] additionally examined the ERI model (average size 60). Further, Chandola and colleagues reported that the results of 16 studies examining work stress and diurnal cortisol patterns were inconclusive (26). Several studies reported gender-specific analysis, showing a positive association between job strain and cortisol levels was more pronounced in women [13], [15], [17]; while over-commitment and ERI were only related to elevated cortisol among men [24], [27].

Inconsistent findings of prior studies could be ascribed to small study sample sizes [26], incomplete information on work stress [27], non-adherence to cortisol sampling protocol (particularly the accuracy of collection time), lack of consistency in modelling cortisol parameters [29], [30] and confounding adjustments [15], [17], [30]. The present study seeks to address these issues by investigating the relation between work stress and diurnal salivary cortisol in a large occupational cohort. We employ the complementary JDC and ERI models, examine cortisol patterns throughout the day and adjust for a variety of covariates. The primary aim of the study was to understand the nature of the relationships between work stress models and indices of diurnal cortisol patterns. A secondary aim was to analyse the gender-specific cortisol pattern by work stress models.

## Methods

### Ethic Statement

Ethical approval for the Whitehall II study was obtained from the University College London Medical School Committees on the Ethics of Human Research. All participants are asked to give written informed consent at each phase.

### Study population

Established in 1985, the Whitehall II study is an on-going cohort with 10,308 participants (66% male, aged 35–55) recruited from 20 London based civil service departments. After the baseline clinical health check-up, further self-administered questionnaire data were collected in follow-up phases administered approximately every two years while repeated clinical examinations were only carried out in odd phases [31]. By Phase 7 (2002–2004) the number of participants was 6,967, half of which were still working (n = 3,413). As the recruitment for the saliva collection was initiated partway through Phase 7, only 65.8% of those working participants (n = 2,246) had information on salivary cortisol. The present analysis focused on participants who were still working in Phase 7, with information on work stress and diurnal cortisol secretion (n = 2,126).

### Measurements of work-stress

The JDC model was assessed by the Job Strain Questionnaire [32]. The questionnaire consisted of three basic components: job demand (4 items, Cronbach's α = 0.67), job control (15 items, Cronbach's α = 0.84) and social support at work (6 items, Cronbach's α = 0.79). A four-point scale from ‘‘often’’ to ‘‘never/almost never’’ were used to answer all these items. Responses were combined into summary scales, where higher scores indicate higher control, demand or support. We used both binary and continuous measurements for job strain. Binary job strain was defined as participants reported both high score on demand (above the median score) and low score on control (below the median score). A continuous scale of job strain was calculated by subtracting control score from demand score. The binary job strain was used to describe participants' characteristics at baseline, and the continuous one was used in regression analysis to prevent any information reduction due to artificial categorizing.

The English version of ERI questionnaires were constructed from the 23 validated Likert scaled items [12], which contained extrinsic and intrinsic dimensions of the full ERI model. For the extrinsic part, effort and reward each was rated on a five-point scale: 4 items for effort (Cronbach's α = 0.80) and 8 items for the reward (Cronbach's α = 0.87). A ratio of ERI was calculated by the formula effort/reward*c [12], where ‘c’ is a correction factor weighting the different numbers of items in numerator and denominator (4/8). ERI>1.0 reflects disproportionate effort, whereas a value from 0 to 1 indicates favourable balance. The continuous ERI ratio was logarithm transformed to produce proportional scaling above and below the balance point “1” [33].

### Cortisol collection and analysis

The protocol of saliva sampling used in the Whitehall II study has been reported previously [34]. Salivettes (Sarstedt, Leicester, UK) were used to collect participants' saliva samples. Participants were instructed to collect 6 samples across the day, at awakening, 30 minutes after waking, 2.5 hours after waking, 8 hours after waking, 12 hours after waking and bedtime. Time of sampling was recorded simultaneously. Participants were required to take samples immediately after awakening. Caffeine and acidic drinks in the first 30 minutes, brushing teeth or eating or drinking 15 minutes before a sample collection were not allowed. Saliva samples were centrifuged at 3000 rpm for 5 minutes. The clear supernatant was assayed via chemiluminescence detection (CLIA; IBL-Hamburg, Hamburg, Germany) to measure the salivary cortisol levels. The lower concentration limit of this assay was 0.44 nmol/l; intra- and inter-assay coefficients of variance were <8%. Any sample >50 nmol/l was repeated.

### Assessment of covariates

Data on gender, age and ethnicity were collected by questionnaires. Waking up time was available from the logbook on the day of sample collection. Time since waking, which shows the time difference between waking and taking first sample was categorized into 5-minute intervals. Social position, assessed by civil service employment grade, was used in this analysis as a potential confounder, given previous studies have described associations with diurnal cortisol patterns [17], [35]. Three categories, administrative (highest employment grade), professional (medium employment grade) and clerical (lowest employment grade), were determined by current civil service employment grade if participants were still working in the civil service or according to the last job grade if participants had left civil service. Body mass index (BMI-kg/m^2^) was categorized using cut-points: <21, 21∼31 and 31+, given a nonlinear association of BMI with diurnal cortisol slope [36].

### Statistical analysis

#### 1. Data reduction for cortisol assessment

Approximately 1% of cortisol values that were three standard deviations above the mean were removed (n = 43), which may be influenced by altered pH-values or blood contamination [29]. Additionally, participants reporting either eating, drinking, exercising or brushing their teeth before the first sample (n = 41) were excluded from the analysis. Data were analysed for difference between weekday/weekend collections. Since no statistically significant differences were observed, data were combined for further analysis. Because of a strong positive skew still existed following removal of outliers, cortisol data were logarithm transformed for analysis.

#### 2. CAR and slope calculation

The CAR was computed as the difference between cortisol values at awaking and 30 minutes after awaking. Conventionally, a delayed sample collected over 10 minutes after awaking is removed due to a reduced CAR [37]. However, we did not find lateness to be significantly associated with work stress. Therefore, instead of excluding those delayed samples, time since waking was included as a covariate.

The methodology used to calculate the slope in cortisol across the day has been previously reported [38]. In short, the slope was derived from regressing cortisol concentration of five samples over the day excluding the second sample, as CAR and slope might be modulated by different neurobiological systems [39]. A multilevel regression model was employed to predict the log cortisol, taking measurement occasion as a level one identifier, person as a level two identifier and sample time as the independent covariate. For each person, the slope was estimated as the overall negative slope plus the level-two slope residual. A more rapid cortisol decline over the day was represented by more negative slope value, whereas flatter diurnal rhythms were indicated as slope values close to zero.

#### 3. Analytic strategy

Participant characteristics and cortisol profile were analysed according to work stress categories using regression analysis for continuous variables and Chi-square test for categorical variables. Linear regression models with CAR or slope as the outcome were employed to assess the association with work stress, adjusted for age, gender, ethnicity, time of waking and time since waking. One at a time, the diurnal cortisol parameters were used as dependent variables and each component of work stress models as independent variables. Since continuous scales were used, results were reported by per standard deviation change in each independent variable. Gender effects were analysed by adding interaction term between gender and work stress. Additional adjustments for employment grade and BMI were run by using multivariable adjusted linear regression models. The data were analysed using STATA version 11.

## Results

### Descriptive results

In Phase 7 half (49.2%) of participants were still working (n = 3,413), 50.8% were not working due to retirement or sickness. Compared with people who were still working, those retired or not working were more likely to be female, older and worked in lower employment grades. The final number of participants for this analysis was 2,126, of whom 481 were female (22.6%). They were more likely to be male, younger and had higher employment grade in comparison with those who were still working in Phase 7 but not included in this analysis (Table 1).

**Table 1:** Participant Characteristics at Whitehall II Phase 7 (2002–2004).

	Participants who attended Phase7(n = 6,967)	Participants still working in Phase7 (n = 3,413)	Participants included in this analysis (n = 2,126)
Male (%)	70.2	75.5	77.4
Mean age (SD)	61.2 (6.0)	57.5 (4.3)	57.1 (4.0)
Ethnic (non-white) (%)	8.2	7.2	6.6
Not married/cohabiting (%)	24.6	21.0	21.6
Lowest employment grade (%)	10.8	8.0	7.0
Body mass index (kg/m^2^) (SD)	26.8 (4.4)	26.8 (4.3)	26.8 (4.3)

SD: standard deviation.

Table 2 shows the characteristics of participants with measures of cortisol secretion stratified by job strain and ERI. In our study sample, the prevalence of job strain was 21% and the prevalence of ERI was 28%. Participants reporting job strain or ERI were younger. Those who reported job strain were less likely to be ethnic minority groups and live with a partner. On the other hand, participants working in higher employment grades were more likely to report ERI. As regards the diurnal cortisol profile, all parameters of cortisol secretion were comparable between either job strain or ERI categories.

**Table 2:** Participant characteristics with data available for work stress and cortisol secretion at Whitehall II Phase 7 (2002–2004) ^#^.

**JDC model**	**No Job Strain (n = 1,653)**	**Job Strain (n = 441)**
Age-mean (sd)	57.4 (4.2)	55.9 (3.1)**
Women (%)	21.9	24.9*
Ethnic (non-white) (%)	7.3	3.4**
Living without partner (%)	20.2	25.9*
Lowest employment grade (%)	7.0	6.6
BMI (kg/m^2^) - mean (sd)	26.8 (4.3)	26.7 (4.4)
CAR (nmol/l) -mean (sd)^b^	7.4 (11.5)	8.1 (11.3)
Slope (nmol/l/hr) -mean (sd)^b^	−0.129 (0.023)	−0.128 (0.023)
**ERI model**	**No ERI (n = 1,501)**	**ERI (n = 589)**
Age-mean (sd)	57.7 (4.2)	55.6 (2.9)**
Women (%)	22.2	23.4
Ethnic (non-white) (%)	6.7	5.9
Living without partner (%)	20.7	22.7
Lowest employment grade (%)	7.7	4.6*
BMI (kg/m^2^) - mean (sd)	26.7 (4.2)	27.0 (4.7)
CAR (nmol/l) -mean (sd)^b^	7.7 (11.6)	7.6 (11.2)
Slope (nmol/l/hr) -mean (sd)^b^	−0.129 (0.023)	−0.128 (0.024)

^#^Within the 2,126 participants included in current analysis, 2,094 and 2,090 had complete data for job strain and ERI measures, respectively. CAR, cortisol awakening response; Slope, cortisol decline across the day.

^b^ Cortisol data adjusted for age, gender, ethnicity.

* P<0.05, ** P<0.01.

### Correlation of work stress measures

The correlation matrix of work stress measures is summarized in Table 3. Demand and effort, support and reward were moderately correlated confirming those measures tapping similar aspects of work stress. Job strain is composed of demand and reward and ERI is composed of effort and reward. Demand had a stronger correlation with job strain than control; the ERI score was highly driven by the effort score. Overall, the directions of those associations confirmed the theoretical assumptions underlying those work stress models, and indicated distinct aspects of work environment may be captured by different dimension of work stress models.

**Table 3:** Correlation matrix for the work stress measures within participants included in analysis.

	control	demand	support	job strain	effort	reward
**demand**	0.20*					
**support**	0.23*	−0.17*				
**job strain**	−0.47*	0.75*	−0.32*			
**effort**	0.08^**^	0.68*	−0.18*	0.56*		
**reward**	0.28*	−0.29*	0.49*	−0.44*	−0.37*	
**ERI ratio**	−0.05**	0.66*	−0.31*	0.57*	0.94*	−0.61*

The spearman rank correlation coefficient (P) are reported

* p<0.001, ** p<0.05

### Relation between work stress and salivary cortisol indices

**Figure 1:**
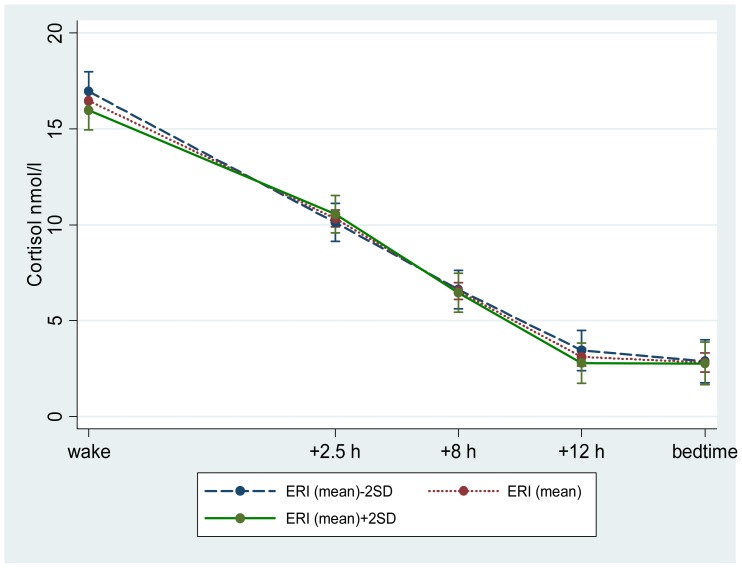
Diurnal cortisol decline by Effort-Reward-Imbalance (ERI) status. Figure 1. Diurnal cortisol decline (adjusted means including 95% CI) by ERI status, adjusted for age, gender, ethnicity, time of waking and time since waking. ERI: effort-reward-imbalance ratio; SD: standard deviation.

**Figure 2:**
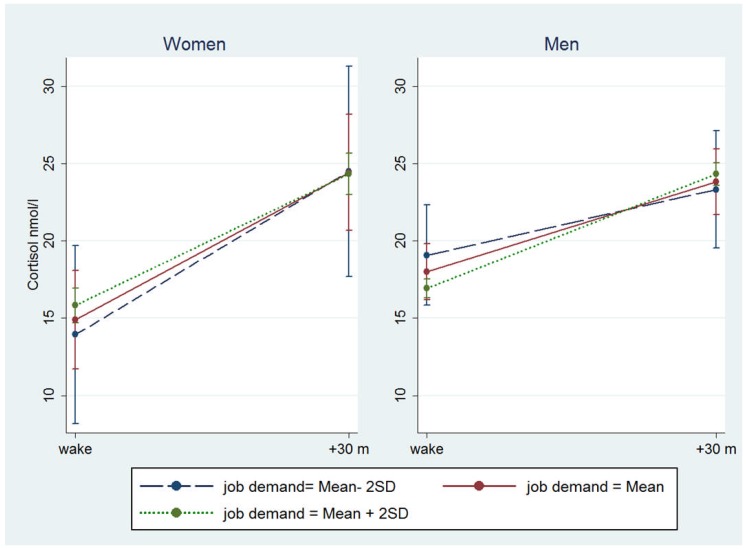
Salivary cortisol levels at waking and 30-min-later by job-demand in women and men. Figure 2. Salivary cortisol levels (adjusted means including 95% CI) at waking and 30-min later by job demand status in women and men, adjusted for age, gender, ethnicity, time of waking and time since waking. SD: standard deviation.

The linear regression is presented in all participants adjusted for gender (Table 4) and gender stratified (Table 5). In all participants, marginally significant associations were only found between slope and the ERI model (Table 4). Lower reward and higher ERI were associated with a shallower slope in cortisol across the day. A shallow slope can be due to depressed morning levels or raised evening levels of cortisol or a combination of both. We therefore assessed the associations with log transformed morning and evening cortisol in relation to work stress. No associations of reward and ERI with morning or evening cortisol were significant, although trends consistent with depressed morning levels and raised evening levels in cortisol were observed (Figure 1). Gender stratified results from linear regression are presented in Table 5. The interaction terms were borderline significant between gender and demand, support and job strain in relation to CAR; while none of the gender interaction terms were significant in cortisol and ERI models. In women, a smaller CAR was associated with higher demand, lower support and higher job strain. A reversed pattern showed in men although not significant (Figure 2).

**Table 4:** Measures of work stress and cortisol secretion measures in all participants at Whitehall II Phase 7, adjusted for age, gender, ethnicity, time of waking and time since waking.

		CAR				Slope		
	N	Coef.	CI	P	N	Coef.	CI	P
**JDC model**								
**Job strain**	1988	0.05	(−0.36,0.82)	0.82	1926	0.0003	(−0.0005,0.0012)	0.36
**control**	1988	−0.10	(−0.61,0.42)	0.71	1926	0.0002	(−0.0009,0.0012)	0.71
**demand**	2003	−0.01	(−0.53,0.52)	0.98	1940	0.0008	(−0.0003,0.0019)	0.14
**Support**	1922	0.14	(−0.37,0.64)	0.61	1863	−0.0004	(−0.0014,0.0007)	0.49
**ERI model**								
**ERI ratio**	1986	−0.33	(−1.56,0.90)	0.61	1922	0.0023	(−0.0002,0.0049)	0.05
**effort**	1988	0.11	(−0.41,0.63)	0.67	1934	0.0007	(−0.0003,0.0018)	0.18
**reward**	1990	0.26	(−0.26,0.78)	0.33	1927	−0.0011	(−0.002,0.00001)	0.04

CAR, cortisol awakening response; Slope, cortisol decline over the day

Data were presented by 1-Standard Deviation increase of each dimension of JDC/ERI models. Job strain was calculated by subtracting control score from demand score; ERI ratio was calculated by the formula effort/reward*0.5 and logarithm transformed.

**Table 5:** Gender-specific associations between measures of work stress and cortisol secretion measures at Whitehall II Phase 7, adjusted for age, ethnicity, time of waking and time since waking.

	CAR					Slope				
	Men		Women		P*	Men		Women		P*
	Coef.	P	Coef.	P		Coef.	P	Coef.	P	
**JDC model**										
**Job strain**	0.24	0.29	−0.75	0.06	0.03	0.0002	0.65	0.0007	0.34	0.55
**control**	−0.14	0.64	0.04	0.94	0.77	0.0002	0.78	0.0003	0.77	0.91
**demand**	0.28	0.35	−1.01	0.06	0.03	0.0006	0.30	0.0014	0.21	0.53
**Support**	−0.14	0.63	1.02	0.05	0.05	−0.0002	0.38	0.0002	0.87	0.57
**ERI Model**										
**ERI ratio**	0.22	0.76	−1.78	0.13	0.14	0.0032	0.03	0.0002	0.95	0.27
**effort**	0.39	0.21	−0.64	0.19	0.08	0.0013	0.05	−0.0007	0.49	0.10
**reward**	0.19	0.57	0.49	0.33	0.58	−0.0012	0.11	−0.0018	0.26	0.88

CAR, cortisol awakening response; Slope, cortisol decline over the day

* : P-value for gender and work stress measurement interaction.

Data were presented by 1-Standard Deviation increase of each dimension of JDC/ERI models. Job strain was calculated by subtracting control score from demand score; ERI ratio was calculated by the formula effort/reward*0.5 and logarithm transformed.

To test the consistency of those relationships, we further adjusted for potential confounding factors. The associations between slope and two components of the ERI model (reward and effort-reward imbalance) remained unchanged after controlling for employment grade. Additional adjustment for BMI had little influence.

## Discussion

This study examined two dominant work stress models and their association with two parameters of the diurnal salivary cortisol pattern. Our results show modest to weak associations between work stress and diurnal cortisol, namely lower reward and higher ERI were related to a flatter diurnal decline. The results also suggest potential gender-specific associations between JDC model and CAR.

The finding of a flatter diurnal cortisol decline was associated with lower reward and higher ERI, is in accordance with some [14], [21], [22], [27] but not all [23], [24]. Our study is considerably larger than previous studies and describes a modest association of work stress with slope in cortisol. The small effect size of this association may explain the lack of consistent findings apparent in the literature, which is mainly composed of small convenience samples and subject to publication bias [1].

We failed to find any significant association in terms of CAR. This is in contrast to the conclusion made in the Chida and Steptoe review (2009). In this review, 22 studies examined work stress and CAR, which included 4 studies that examined the JDC and/or ERI model [13], [23], [27], [28]. In comparison to our study these studies were small (pooled sample size: 637), but had the advantage that participants were younger. The absent association of work stress and CAR may be owing to a low prevalence of work stress in current study. The prevalence of job strain was 21%, within the low range of 10%–40% reported by Siegrist [7]. This low prevalence may be due to early retirement of those had experienced work stress, and therefore were not included in the analysis. Further, remaining participants were more likely from higher employment grades with more control power. Given the relatively low reliability of the demand measure, the effect of job demand may be underestimated, which in turn may result in an underestimated job strain driven by high control score. Moreover, the participants in the current study were in the pre-retirement phase of their working life, and this may be a stage in the lifecourse when stressors associated with home life are more pertinent than work stress per se [40]–[42].

The moderate associations apparent in our study are difficult to explain and may relate to the cross-sectional nature of the analysis and the age of our participants. According to the stress response theory, only those prolonged stressful conditions which involve uncontrollable, social-evaluative and unpredictable elements can significantly affect the magnitude of cortisol response and time to recovery [43], [44]. Therefore routine work-related stressors with low perceived level of pressure may not be severe enough to evoke a detectable disturbance in cortisol secretion considering the breadth of inter-individual differences [45]–[47]. On the other hand, our results may indicate HPA axis responsiveness had been adapted to chronic stress in this group of older participants, such that there was a lower rather than higher stress response [7], [48]. Evidence shows that an impaired feedback regulation of the HPA axis may underlie the flatter diurnal cortisol patters [49], [50], which were associated with fatigue [38] and increased risk of al-cause mortality [51]. However, it was not possible to examine chronic work stress in the analyses as work stress has not been measured in the same way across phases of data collection in the study.

It is possible there is a gender-specific cortisol stress response: ERI appears to be a risk factor in men only [24], [27], whereas JDC appears more relevant to women [13], [15], [17]. Our results contribute to evidence on the JDC model, suggesting women may be more sensitive to job strain with regards to CAR. However, as women only constitute 22.6% of the sample, there is limited power to detect gender differences. Given the high correlation between demand and job strain models, those few marginally significant gender interaction terms should be interpreted with caution.

### Strengths and limitations

The main strength and limitations of our study needed to be discussed. The accurate measures of the main variables strengthened the confidence of our findings. The data on work stress are detailed and comprehensive since the Whitehall II study was established to examine the associations of psychosocial work environment and adverse health consequences. Diurnal cortisol data were collected repeatedly throughout a weekday on a large scale. Besides a high response rate (90.1%), indicators also showed that participants correctly followed the instructions and took salivary samples accordingly (95.5% participants had complete data for 6 samples).

The weaknesses of the current analysis are, first, at Phase 7, 49.2% participants were retired and retirees were more likely to come from lower employment grades, potentially depleting the sample of working men and women with high perceptions of work stress. Further, given the pre-retirement feature of our participants, the association of work stress and cortisol secretion may be underestimated. Second, since the study sample is comprised of white-collar civil servants, the results may not generalise to manual occupations. Nevertheless, the cohort covers a wide occupational spectrum with salary difference more than 10-fold between the top and bottom of the socioeconomic hierarchy. Third, we used a cross-sectional design in order to detect the concurrent biological stress effect; however, this means that the causal direction of the associations observed is unclear. Fourth, as salivary cortisol samples were only collected on a single day, the intra-individual variation could bias the CAR to situational predictors [52]. However, we speculate that this should serve to increase our risk of finding an association of the CAR with concurrently assessed work stress. Last, as a male-dominated cohort, the power to detect gender interactions is low.

In conclusion, this study analysed two complementary work stress models and their associations with diurnal cortisol patterns with regards to gender. Results suggest little evidence of a strong association between work stress and diurnal cortisol in this ageing occupational cohort, such that only the ERI model was moderately related to cortisol diurnal decline. Further studies are needed to confirm potential gender-specific effects of work stress models.

## References

[1] Kivimäki M, Nyberg ST, Batty GD, Fransson EI, Heikkilä K, et al.. (2012) Job strain as a risk factor for coronary heart disease: a collaborative meta-analysis of individual participant data. The Lancet.

[2] Chandola T, Brunner E, Marmot M (2006) Chronic stress at work and the metabolic syndrome: prospective study. Bmj 332: 521–525. 16428252 10.1136/bmj.38693.435301.80 PMC1388129

[3] Gimeno D, Tabák ÁG, Ferrie JE, Shipley MJ, De Vogli R, et al (2010) Justice at work and metabolic syndrome: the Whitehall II study. Occupational and environmental medicine 67: 256–262. 19819861 10.1136/oem.2009.047324 PMC3226946

[4] Heraclides AM, Chandola T, Witte DR, Brunner EJ (2011) Work Stress, Obesity and the Risk of Type 2 Diabetes: Gender-Specific Bidirectional Effect in the Whitehall II Study. Obesity(Silver Spring). 10.1038/oby.2011.95 21593804

[5] Kumari M, Head J, Marmot M (2004) Prospective study of social and other risk factors for incidence of type 2 diabetes in the Whitehall II study. Arch Intern Med 164: 1873–1880. 15451762 10.1001/archinte.164.17.1873

[6] Karasek Jr RA (1979) Job demands, job decision latitude, and mental strain: Implications for job redesign. Administrative science quarterly: 285–308.

[7] Siegrist J (1996) Adverse health effects of high-effort/low-reward conditions. Journal of Occupational Health Psychology 1: 27. 9547031 10.1037//1076-8998.1.1.27

[8] Harma M, Kompier MAJ, Vahtera J (2006) Work-related stress and health-risks, mechanisms and countermeasures. Scandinavian Journal of Work Environment and Health 32: 413. 10.5271/sjweh.1047 17173198

[9] Bosma H, Peter R, Siegrist J, Marmot M (1998) Two alternative job stress models and the risk of coronary heart disease. American Journal of Public Health 88: 68. 9584036 10.2105/ajph.88.1.68 PMC1508386

[10] Elovainio M, Ferrie JE, Singh-Manoux A, Gimeno D, De Vogli R, et al (2009) Cumulative exposure to high-strain and active jobs as predictors of cognitive function: the Whitehall II study. Occupational and environmental medicine 66: 32–37. 18805883 10.1136/oem.2008.039305 PMC2740874

[11] Van Vegchel N, De Jonge J, Bosma H, Schaufeli W (2005) Reviewing the effort-reward imbalance model: drawing up the balance of 45 empirical studies. Social Science & Medicine 60: 1117–1131. 15589679 10.1016/j.socscimed.2004.06.043

[12] Siegrist J, Starke D, Chandola T, Godin I, Marmot M, et al (2004) The measurement of effort-reward imbalance at work: European comparisons. Social Science & Medicine 58: 1483–1499. 14759692 10.1016/S0277-9536(03)00351-4

[13] Alderling M, Theorell T, De La Torre B, Lundberg I (2006) The demand control model and circadian saliva cortisol variations in a Swedish population based sample (The PART study). BMC Public Health 6: 288. 17129377 10.1186/1471-2458-6-288 PMC1693564

[14] Maina G, Bovenzi M, Palmas A, Larese Filon F (2009) Associations between two job stress models and measures of salivary cortisol. International archives of occupational and environmental health 82: 1141–1150. 19554345 10.1007/s00420-009-0439-0

[15] Maina G, Palmas A, Bovenzi M, Filon FL (2009) Salivary cortisol and psychosocial hazards at work. American journal of industrial medicine 52: 251–260. 19023870 10.1002/ajim.20659

[16] Steptoe A, Cropley M, Griffith J, Kirschbaum C (2000) Job strain and anger expression predict early morning elevations in salivary cortisol. Psychosomatic Medicine 62: 286–292. 10772410 10.1097/00006842-200003000-00022

[17] Kunz-Ebrecht SR, Kirschbaum C, Steptoe A (2004) Work stress, socioeconomic status and neuroendocrine activation over the working day. Social Science & Medicine 58: 1523–1530. 14759695 10.1016/S0277-9536(03)00347-2

[18] Steptoe A, Wardle J, Lipsey Z, Mills R, Oliver G, et al (1998) A longitudinal study of work load and variations in psychological well-being, cortisol, smoking, and alcohol consumption. Annals of Behavioral Medicine 20: 84–91. 9989313 10.1007/BF02884453

[19] Dahlgren A, Kecklund G, Akerstedt T (2005) Different levels of work-related stress and the effects on sleep, fatigue and cortisol. Scandinavian Journal of Work Environment and Health 31: 277. 10.5271/sjweh.883 16161710

[20] Fujiwara K, Tsukishima E, Kasai S, Masuchi A, Tsutsumi A, et al (2004) Urinary catecholamines and salivary cortisol on workdays and days off in relation to job strain among female health care providers. Scandinavian journal of work, environment & health 30: 129–138. 10.5271/sjweh.770 15127783

[21] Bellingrath S, Kudielka BM (2008) Effort-reward-imbalance and overcommitment are associated with hypothalamus-pituitary-adrenal (HPA) axis responses to acute psychosocial stress in healthy working schoolteachers. Psychoneuroendocrinology 33: 1335–1343. 18774231 10.1016/j.psyneuen.2008.07.008

[22] Bellingrath S, Weigl T, Kudielka BM (2008) Cortisol dysregulation in school teachers in relation to burnout, vital exhaustion, and effort-reward-imbalance. Biological psychology 78: 104–113. 18325655 10.1016/j.biopsycho.2008.01.006

[23] Harris A, Ursin H, Murison R, Eriksen HR (2007) Coffee, stress and cortisol in nursing staff. Psychoneuroendocrinology 32: 322–330. 17350175 10.1016/j.psyneuen.2007.01.003

[24] Steptoe A, Siegrist J, Kirschbaum C, Marmot M (2004) Effort—reward imbalance, overcommitment, and measures of cortisol and blood pressure over the working day. Psychosomatic Medicine 66: 323–329. 15184690 10.1097/01.psy.0000126198.67070.72

[25] Chida Y, Steptoe A (2009) Cortisol awakening response and psychosocial factors: a systematic review and meta-analysis. Biol Psychol 80: 265–278. 19022335 10.1016/j.biopsycho.2008.10.004

[26] Chandola T, Heraclides A, Kumari M (2010) Psychophysiological biomarkers of workplace stressors. Neurosci Biobehav Rev 35: 51–57. 19914288 10.1016/j.neubiorev.2009.11.005 PMC2891393

[27] Eller NH, Netterstrøm B, Hansen ÅM (2006) Psychosocial factors at home and at work and levels of salivary cortisol. Biological psychology 73: 280–287. 16824664 10.1016/j.biopsycho.2006.05.003

[28] Sjögren E, Leanderson P, Kristenson M (2006) Diurnal saliva cortisol levels and relations to psychosocial factors in a population sample of middle-aged Swedish men and women. International Journal of Behavioral Medicine 13: 193–200. 17078769 10.1207/s15327558ijbm1303_2

[29] Kunz-Ebrecht SR, Kirschbaum C, Marmot M, Steptoe A (2004) Differences in cortisol awakening response on work days and weekends in women and men from the Whitehall II cohort. Psychoneuroendocrinology 29: 516–528. 14749096 10.1016/s0306-4530(03)00072-6

[30] Hjortskov N, Garde AH, Ørbæk P, Hansen ÅM (2004) Evaluation of salivary cortisol as a biomarker of self-reported mental stress in field studies. Stress and health 20: 91–98.

[31] Marmot M, Brunner E (2005) Cohort profile: the Whitehall II study. International journal of epidemiology 34: 251–256. 15576467 10.1093/ije/dyh372

[32] Bosma H, Marmot MG, Hemingway H, Nicholson AC, Brunner E, et al (1997) Low job control and risk of coronary heart disease in Whitehall II (prospective cohort) study. Bmj 314: 558. 9055714 10.1136/bmj.314.7080.558 PMC2126031

[33] Pikhart H, Bobak M, Pajak A, Malyutina S, Kubinova R, et al (2004) Psychosocial factors at work and depression in three countries of Central and Eastern Europe. Social Science & Medicine 58: 1475–1482. 14759691 10.1016/S0277-9536(03)00350-2

[34] Badrick E, Kirschbaum C, Kumari M (2007) The relationship between smoking status and cortisol secretion. Journal of Clinical Endocrinology & Metabolism 92: 819–824. 17179195 10.1210/jc.2006-2155

[35] Kumari M, Badrick E, Chandola T, Adler NE, Epel E, et al (2010) Measures of social position and cortisol secretion in an aging population: findings from the Whitehall II study. Psychosomatic Medicine 72: 27–34. 19995885 10.1097/PSY.0b013e3181c85712

[36] Kumari M, Chandola T, Brunner E, Kivimaki M (2010) A nonlinear relationship of generalized and central obesity with diurnal cortisol secretion in the Whitehall II study. Journal of Clinical Endocrinology & Metabolism 95: 4415–4423. 20591984 10.1210/jc.2009-2105 PMC2936066

[37] Kudielka BM, Schommer NC, Hellhammer DH, Kirschbaum C (2004) Acute HPA axis responses, heart rate, and mood changes to psychosocial stress (TSST) in humans at different times of day. Psychoneuroendocrinology 29: 983–992. 15219648 10.1016/j.psyneuen.2003.08.009

[38] Kumari M, Badrick E, Chandola T, Adam EK, Stafford M, et al (2009) Cortisol secretion and fatigue: Associations in a community based cohort. Psychoneuroendocrinology 34: 1476–1485. 19497676 10.1016/j.psyneuen.2009.05.001

[39] Clow A, Thorn L, Evans P, Hucklebridge F (2004) The awakening cortisol response: methodological issues and significance. Stress: The International Journal on the Biology of Stress 7: 29–37. 10.1080/10253890410001667205 15204030

[40] Hyde M, Ferrie J, Higgs P, Mein G, Nazroo J (2004) The effects of pre-retirement factors and retirement route on circumstances in retirement: findings from the Whitehall II study. Ageing and Society 24: 279–296.

[41] Sutinen R, Kivimäki M, Elovainio M, Forma P (2005) Associations between stress at work and attitudes towards retirement in hospital physicians. Work & Stress 19: 177–185.

[42] Kubicek B, Korunka C, Hoonakker P, Raymo JM (2010) Work and family characteristics as predictors of early retirement in married men and women. Research on aging 32: 467–498. 21430790 10.1177/0164027510364120 PMC3061466

[43] Dickerson SS, Kemeny ME (2004) Acute stressors and cortisol responses: a theoretical integration and synthesis of laboratory research. Psychological bulletin 130: 355. 15122924 10.1037/0033-2909.130.3.355

[44] Kudielka BM, Kirschbaum C (2005) Sex differences in HPA axis responses to stress: a review. Biological psychology 69: 113–132. 15740829 10.1016/j.biopsycho.2004.11.009

[45] Kajantie E, Phillips DIW (2006) The effects of sex and hormonal status on the physiological response to acute psychosocial stress. Psychoneuroendocrinology 31: 151–178. 16139959 10.1016/j.psyneuen.2005.07.002

[46] Hanson EKS, Maas CJM, Meijman TF, Godaert GLR (2000) Cortisol secretion throughout the day, perceptions of the work environment, and negative affect. Annals of Behavioral Medicine 22: 316–324. 11253443 10.1007/BF02895668

[47] Karp A, Kåreholt I, Qiu C, Bellander T, Winblad B, et al (2004) Relation of education and occupation-based socioeconomic status to incident Alzheimer's disease. American journal of epidemiology 159: 175–183. 14718220 10.1093/aje/kwh018

[48] Heim C, Ehlert U, Hellhammer DH (2000) The potential role of hypocortisolism in the pathophysiology of stress-related bodily disorders. Psychoneuroendocrinology 25: 1–35. 10633533 10.1016/s0306-4530(99)00035-9

[49] Spiegel D, Giese-Davis J, Taylor CB, Kraemer H (2006) Stress sensitivity in metastatic breast cancer: analysis of hypothalamic-pituitary-adrenal axis function. Psychoneuroendocrinology 31: 1231–1244. 17081700 10.1016/j.psyneuen.2006.09.004 PMC1790857

[50] Gunnar MR, Vazquez DM (2001) Low cortisol and a flattening of expected daytime rhythm: Potential indices of risk in human development. Development and Psychopathology 13: 515–538. 11523846 10.1017/s0954579401003066

[51] Kumari M, Shipley M, Stafford M, Kivimaki M (2011) Association of diurnal patterns in salivary cortisol with all-cause and cardiovascular mortality: findings from the Whitehall II study. Journal of Clinical Endocrinology & Metabolism 96: 1478–1485. 21346074 10.1210/jc.2010-2137 PMC3085201

[52] Hellhammer J, Fries E, Schweisthal O, Schlotz W, Stone A, et al (2007) Several daily measurements are necessary to reliably assess the cortisol rise after awakening: state-and trait components. Psychoneuroendocrinology 32: 80–86. 17127010 10.1016/j.psyneuen.2006.10.005

